# Community-Based Participatory Intervention Research with American Indian Communities: What is the State of the Science?

**DOI:** 10.1093/cdn/nzz008

**Published:** 2019-02-26

**Authors:** Jordan B Hearod, Marianna S Wetherill, Alicia L Salvatore, Valarie Blue Bird Jernigan

**Affiliations:** University of Oklahoma Health Sciences Center, Tulsa, OK

**Keywords:** community-based participatory research, CBPR, systematic review, American Indian, Native American, intervention science

## Abstract

We conducted a 2-phase systematic review of the literature to examine the nature and outcomes of health research using a community-based participatory research (CBPR) approach with AI communities to assess both the value and the impact of CBPR, identify gaps in knowledge, and guide recommendations for AI research agendas. Using PRISMA guidelines, we searched the peer-reviewed literature published from 1995 to 2016 and identified and reviewed 42 unique intervention studies. We identified and catalogued key study characteristics, and using the Reliability-Tested Guidelines for Assessing Participatory Research Projects, we quantified adherence to participatory research principles across its four domains. Finally, we examined any association between community participation score and health outcomes. The majority of studies (76.7%) used an observational study design with diabetes, cancer, substance abuse, and tobacco being the most common topics. Half of the articles reported an increase in knowledge as the primary outcome. Our findings suggest that a CBPR orientation yields improved community outcomes. However, we could not conclude that community participation was directly associated with an improvement in health outcomes.

## Introduction

Community-based participatory research (CBPR) is a partnership approach to research that equitably involves both researchers and community members in all aspects of the research process (1). An alternative to traditional research, CBPR has the added benefit of increasing community capacity, progressing knowledge transfer between partners, and facilitating the *real-world* translation of research into practice within a community context (2–8).

As CBPR has “moved from the margins to the mainstream” of research, the use of this orientation in health research with American Indians (AI) has increased as well. Indeed, the principles of CBPR, such as promoting colearning and achieving a balance between research and action (9–13), in many ways mirror those set forth by AI communities (14). A number of tribal nations have developed their own Institutional Review Boards (IRBs), and federal funding agencies have increasingly called for a CBPR orientation when conducting research with AI peoples, as noted in 1 recent funding announcement from the NIH, “Interventions for Health Promotion and Disease Prevention in Native American Populations” (PAR 11-346; PAR 14-260) that specifically called for and cited the importance of a CBPR approach when working with sovereign tribal nations.

Several systematic reviews document the value of a CBPR orientation (15–19). One of the most comprehensive reviews conducted by Viswathan et al. (2004) found that the use of a CBPR orientation was associated with improved research quality and improved community research capacity, but there was insufficient evidence to conclude if a CBPR approach was associated with improved health outcomes or cost savings. Although the Viswathan et al. review (16) and others included studies conducted with indigenous populations, these reviews did not examine unique circumstances that exist in sovereign tribal nations. For example, the diverse research protocols in place across more than 550 federally recognized tribal nations in the United States often weigh the risks and benefits of proposed research on tribal lands and with tribal community members in the context of tribal culture and community. Some tribes may require ownership of all data collected and often mandate tribal review and approval of all publications, differentiating research conducted with citizens of sovereign tribal nations from research with other racial or ethnic groups.

Though the number of articles published citing a CBPR approach to health research with tribal communities has increased dramatically over the last 2 decades, to our knowledge, no comprehensive review examining the nature and outcomes of CBPR with tribal communities has been published (14). A synthesis of the current literature is needed to better understand how researchers are implementing CBPR. This can be useful for not only advancing the science of CBPR processes, but ultimately the implementation of health-related interventions that better address root determinants of AI health inequities, such as nutrition, healthy food access, and food sovereignty. Thus, this systematic review aimed to comprehensively identify and describe studies using a CBPR approach to health research with indigenous communities in the US. Then, using the Reliability-Tested Guidelines for Assessing Participatory Research Projects (17), we quantified community participation across 4 domains (**Table 1**) and assessed if a higher community participation score was associated with improved health outcomes.

**TABLE 1:** Summary of reliability-tested guidelines^1^ for assessing participatory research projects

Domain 1: Participants and the Nature of Their Involvement	
1a	Are the intended users (may include users, beneficiaries, and/or stakeholders) of the research described adequately enough to assess their representation in the project?
1b	Is the mix of participants included in the research process sufficient to consider the needs of the project's intended users?
1c	Is effort made to address barriers to participation in the research process by intended users who might otherwise tend to be under-represented?
1d	Has provision been made to build trust between researchers and intended users participating in the research process?
1e	Do the researchers and intended users participating in the research process have a formal or informal agreement (verbal or written) regarding management of the project?
Domain 2: Shaping the Purpose and Scope of the Research	
2a	Was (were) the research question(s) developed (or refined) through a collaborative process between researchers and intended users?
2b	Has the proposed research project applied the knowledge and experience of intended users in conceptualizing and/or designing the research?
2c	Does the proposed research project provide for mutual learning among intended users and researchers?
2d	Does the proposed research project consider multiple levels of determinants of health (for example, individual, familial, organizational, political, social, and/or economic)?
2e	Does the proposed research project plan to build the capacity of intended users to address individual or broader determinants of health?
Domain 3: Research Implementation and Context	
3a	Does the proposed research project apply the knowledge and experience of intended users in the implementation of the research?
3b	Does the proposed research project provide intended users participating in the research process with opportunity to learn about research (whether or not the intended users choose to take that opportunity)?
3c	Does the proposed research project provide researchers with opportunity to learn about user perspectives on the issue(s) being studied?
3d	Do the researchers and intended users participating in the research process have a formal or informal agreement (verbal or written) regarding mutual decision-making about potential changes in research methods or focus?
3e	Does the proposed research project provide intended users with opportunity to participate in planning and executing the data collection (whether or not the intended users choose to take that opportunity)?
3f	Does the proposed research project provide intended users with opportunity to participate in planning and/or executing the analysis (whether or not the intended users choose to take that opportunity)?
3g	Are plans to involve intended users in interpreting the research findings sufficient to reflect knowledge of the particular context and circumstances in the interpretation?
Domain 4: Nature of the Research Outcomes	
4a	Does the proposed research project reflect sufficient commitment by researchers and intended users participating in the research process to action (for example, social, individual, and/or cultural) following the (learning acquired through) research?
4b	Do the researchers and intended users engaged in the research process have a formal or informal agreement (verbal or written) for acknowledging and resolving in a fair and open way any differences in the interpretation of research results?
4c	Do the researchers and intended users engaged in the research process have a formal or informal agreement (verbal or written) regarding ownership and sharing of the research data?
4d	Do the researchers and intended users engaged in the research process have a formal or informal agreement (verbal or written) regarding feedback of research results to intended users?
4e	Do the researchers and intended users engaged in the research process have a formal or informal agreement (verbal or written) regarding the dissemination (and/or translation or transfer) of research findings?
4f	Does the proposed research project provide intended users with opportunity to participate in dissemination of project findings to other intended users and researchers (whether or not the intended users choose to take that opportunity)?
4g	Is there sufficient provision for assistance to intended users to indicate a high probability of research results being applied?
4h	Does the proposed research project plan for sustainability in relation to the purpose of the research (for example, by fostering collaboration between intended users and resource providers, funding sources, policymakers, holders of community assets, and the like)?

^1^Original guidelines from Mercer et al. (17).

## Methods

### Search and sampling strategy

**FIGURE 1:**
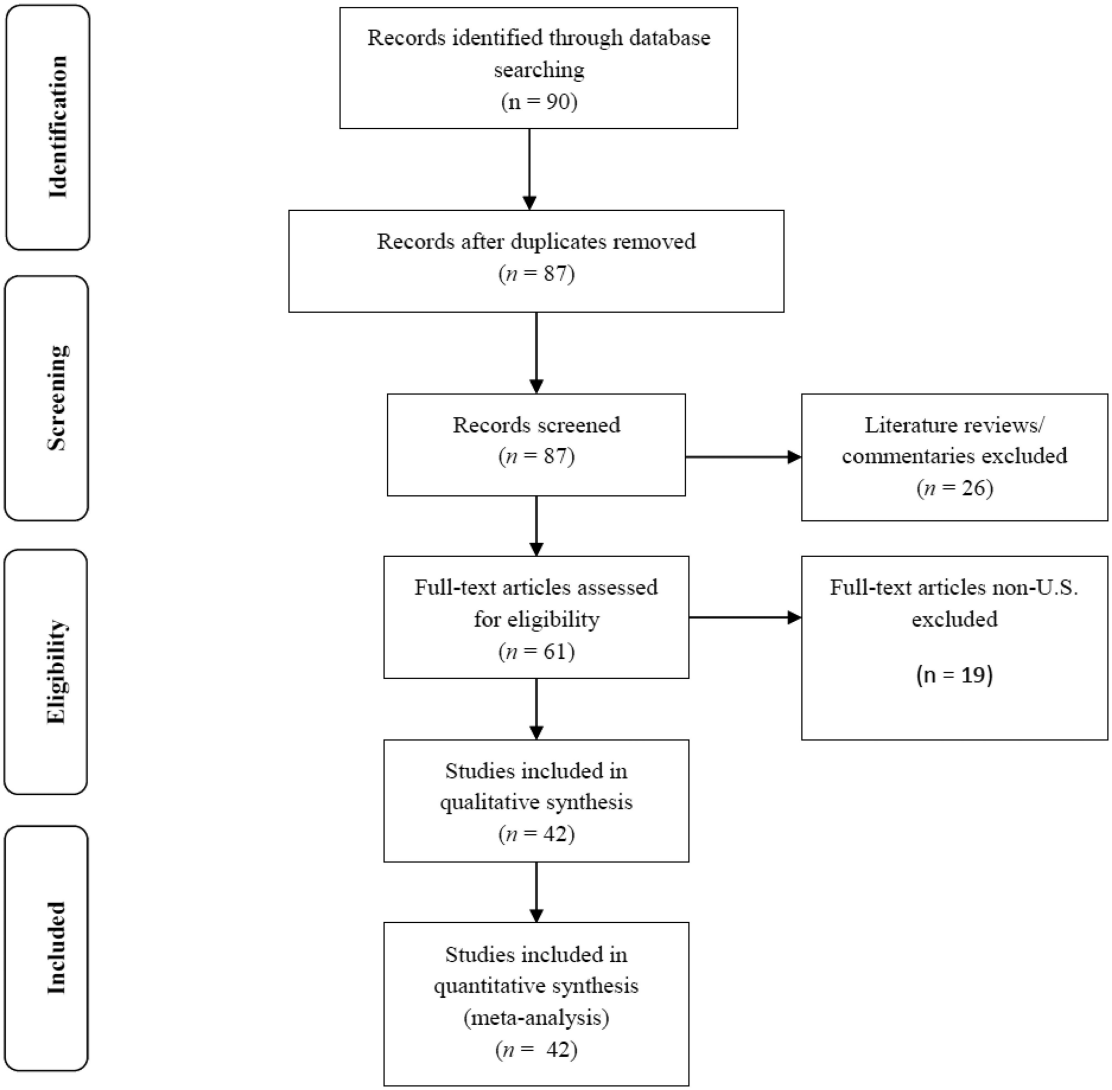
Flow diagram of search and sampling strategy. Reviewers initially identified 90 studies through targeted searches using PubMed, MEDLINE, and Ovid databases for peer-reviewed articles were written in English, published in peer-reviewed journals between January 1995 and February 2016, and reported on public health interventions or programs that used a CBPR orientation in partnership with an AI community in the US. Of the 90 articles, 26 articles that were literature reviews or commentaries and did not describe or discuss a specific intervention study or program with outcomes were excluded. Three articles that reported duplicate methods were excluded. Eighteen articles that were conducted outside the US or with First Nations communities were excluded. The total sample included 42 unduplicated articles. AI, American Indian; CBPR, community-based participatory research.

We searched PubMed, MEDLINE, and Ovid databases for peer-reviewed articles that were written in English, published in peer-reviewed journals between January 1995 and February 2016, and reported on public health interventions or programs that used a CBPR orientation in partnership with an AI community in the US. As such, we only included articles with tribal communities residing in an Indian Health Service (HIS) Region, a common way to categorize AI population or community data (18). We included the following keywords or phrases: *American Indian* OR *Native American* AND *community-based participatory research* OR *CBPR* OR *PAR* OR *participatory research* OR community*-driven research* OR *action science*. This preliminary search yielded 90 articles. Of these 90 articles, we excluded 26 articles that were literature reviews or commentaries and did not describe or discuss a specific intervention study or program with outcomes. We excluded 3 articles that reported duplicate methods (i.e., we already had included publications from these studies that described their CBPR orientation). Lastly, we excluded 18 articles that were conducted outside the US or with First Nations communities. Thus, our total sample included 42 unduplicated articles (**Figure 1**).

### Identification of key study characteristics

We then identified key characteristics for each of the 42 articles within our study (**Table 2**). First, we identified the author and year each study was published. Next, we assessed the study location, which we categorized by Indian Health Service (IHS) Regions. Then, we summarized the topic of each study by disease category (e.g., cancer, diabetes), identified the research design, and summarized any outcomes listed in the reviewed articles. We also assessed the total number of research participants for each study. Then, we identified the funding source for each study. In terms of authorship, we examined whether each article listed a community partner as a coauthor and used a positive determination (i.e., a community coauthored the article) as an indicator of community participation. Lastly, we reviewed whether tribal IRBs or the equivalent bodies (e.g., a community research committee or a regional IHS IRB) had reviewed and approved the study and its publication. We used a positive determination as an indicator for research capacity.

**TABLE 2:** Summary of study characteristics

First author (year)	IHS region	Study topic	Research design	Outcomes	Research participants (*n*)	Funding type (source)	Listed as coauthor	Tribal IRB/equivalent approved
Arviso (2012)	Southwest	Wellness (Youth)	Curriculum Development	Curriculum developed; increase in knowledge, capacity	Navajo educators (NA)	Private (Ford Foundation)	No	No
Baldwin (1998)	Southwest	Substance Abuse and HIV, Prevention (Youth)	Curriculum Development	Participants remained in or moved to lower risk categories; lower rates of sexually risky behavior	Adolescents (2704)	Federal (NIAAA^1^)	No	Yes
Brown (2012)	Northern Plains	Diabetes, Prevention (Youth)	Observational—Qualitative	Increased community capacity; adaptation of a youth diabetes prevention program	Participants aged 10–68 (31)	Federal (NIDDK^2^)	No	No
Christopher (2008)	Northern Plains	Cancer, Screening (Cervical)	Observational— Mixed	Increased knowledge about cervical cancer and Pap tests; increased comfort discussing cancer issues	Adult women (101)	Federal (NA)	Yes	Yes
Coe (2006)	Southwest	Cancer, Prevention, Screening	Observational—Qualitative	Increased community research capacity	Patients at 2 medical centers (NA)	Federal (NCI^3^)	No	Yes
Colclough (2013)	East	End-of-Life Decisions	Observational—Qualitative	Factors for improving end-of-life experiences identified	Persons with end-of-life experience (58)	Federal (NIMHD^4^), Private (Livestrong Foundation), University	No	Yes
Daley (2010)	East/Southern Plains	Cancer, Screening (Colorectal and Breast)	Observational— Qualitative	Increased validity of results due to better recruitment efforts; increased participation in screening	Adults living in Kansas and Missouri (519)	Federal (NCI^3^), Private (ACS^5^, ALA^6^, Susan G. Komen Foundation)	No	No
Fahrenwald (2010)	Northern Plains	Organ Donation	Observational—Quantitative	Change in knowledge, attitudes, and beliefs about organ donation	Adults from 4 rural tribes (1580)	Federal (HHS^7^, HRSA^8^)	Yes	Yes
Filippi (2013)	East/Southern Plains	Screening, Cancer (Breast)	Observational— Qualitative	Perceptions of breast cancer screening/barriers and suggestions to improve access documented	Women aged 25–39 (48)	Federal (NIMHD^4^)	No	Yes
Fleischacker (2012)	East	Nutrition, Healthy Eating	Observational—Mixed	Toolkit developed; increase in knowledge and awareness regarding engagement and tailoring	Adults (77)	Federal (NIH^9^), Private (RWJF^10^)	No	No
Forcehimes (2011)	Southwest	Substance Abuse	Observational— Mixed	Methamphetamine and alcohol identified as community health concerns	Community members (481)	Federal (NIDA^11^)	Yes	Yes
Gilder (2013)	Pacific Coast	Substance Abuse (Youth)	Observational—Quantitative	Community research capacity built; colearning promoted	Registered patients aged 8–20 (197)	Federal (NIAAA^1^, NIMHD^4^)	No	Yes
Goins (2011)	East	Elder Health	Observational—Qualitative	Increase in knowledge regarding elder care	Elders over 65 (NA)	Federal (NIH^9^)	Yes	Yes
Guadagnolo (2009)	Northern Plains	Cancer, Screening (All Types)	Observational—Quantitative	Change in knowledge and attitudes regarding cancer screening	AI/non-AI cancer patients (165)	Federal	No	Yes
Haozous (2010)	Southern Plains	Cancer, Screening (Breast)	Observational— Qualitative	Increase in knowledge regarding barriers and perceptions of breast cancer screening	Community Health Representatives (7)	University	No	Yes
Hartman (2012)	NA	Mental Health, Traditional Healing as Treatment	Observational— Qualitative	Increase in knowledge regarding the relation of mental health and traditional healing	Urban adults (26)	University	No	Yes
Helitzer (2009)	Southwest	Injury Prevention	Observational— Qualitative	Logic model to improve knowledge, attitudes, and behaviors about integrated pest management created	Navajo community members (NA)	Federal (NIOSH^12^, CDC^13^)	No	Yes
Horn (2008)	East	Tobacco, Cessation	Curriculum Development	Creation and adaptation of tools; trust built; needs identified	Teens (74)	Federal (CDC^13^), Private (ALF^14^)	No	No
Jernigan (2012)	Pacific Coast	Nutrition, Food Insecurity	Observational— Qualitative	Producers’ Guild formed; CSA program developed; EBT machines placed at farmer's market; shelf space in grocery stores reallocated; walking/biking path designed with culturally appropriate art	Adults (40)	Private Foundation (RWJF^10^), State (CA Endowment, CA Department of Transportation)	No	Yes
Katz (2011)	Pacific Coast	Substance Abuse, Prevention (Youth)	Observational— Qualitative	Strengthened partnership between university and community	Individuals aged 12–99 (95)	NA	No	Yes
Lopez (2012)	Alaska	Education (College Attrition)	Observational— Mixed	Creation of culturally tailored tools; community empowered; capacity built	College students (NA)	Federal (NCRR^15^)	Yes	No
Lowe (2012)	Southern Plains	Substance Abuse, Prevention (Youth)	Curriculum Development	Reduction in substance abuse	Adolescents (187)	Federal (NIDA)	Yes	Yes
Markus (2012)	Northern Plains	STDs/HIV, Prevention (Youth)	Observational— Qualitative	Increase in knowledge regarding HIV/STIs; empowerment of youth	Youth aged 18–19 (6)	State (WY)	No	No
Matloub (2009)	Northern Plains	Cancer, Surveillance	Observational—Quantitative	Change in knowledge regarding cancer; relations built	De-identified medical records (NA)	Federal (NCI^3^)	No	Yes
Mendenhall (2010)	Northern Plains	Diabetes, Screening	Observational—Quantitative	Themes for improving Family Education Diabetes Series generated	Adults (32)	NA	Yes	No
Montgomery (2012)	Pacific Coast	Tobacco, Prevention (Youth)	Curriculum Development	Increase in knowledge regarding tobacco; integration of community concepts	Youth aged 12–15 (6)	Federal (NCI^3^, NCRR^15^)	No	No
Mullany (2009)	Southwest	Suicide (Youth)	Observational—Quantitative	Suicide prevention program developed	Adults aged < 25 y (41)	Federal (NIGMS^16^, IHS^17^)	Yes	Yes
Mullany (2012)	Southwest	Pregnancy, Tobacco	Experimental	Increase in knowledge regarding tobacco	Pregnant teen mothers (322)	Federal (NIDA^11^)	No	Yes
Ndikum-Moffor (2013)	East/Southern Plains	Cancer, Screening (Breast)	Observational— Qualitative	Community themes for mammography improvement generated	Women ≥ 40 (53)	Federal (NIMHD^4^)	No	Yes
Noe (2007)	Northern Plains	Health Behavior Change	Observational—Quantitative	Principles of CBPR shown to be effective in changing health behaviors	College students (1066)	NA	No	Yes
Novins (2012)	Southern Plains	Substance Abuse (Youth)	Curriculum Development	Pilot study showed feasibility in reducing substance abuse	Clinicians, administrators, youth, parents, elders, and researchers (NA)	Federal (NIMH^18^)	Yes	No
Nsiah-Kumi (2012)	NA	Diabetes, Screening (Youth)	Observational—Quantitative	Increase in knowledge regarding diabetes	Youth aged 5–18 (201)	Federal (NIDDK^2^)	Yes	Yes
Palacios (2013)	NA	Maternal/Child Health, Breastfeeding (Youth)	Observational— Qualitative	Increase in knowledge regarding culturally appropriate nursing techniques	Teen mothers (30)	Federal (NIH^9^), University	No	Yes
Perry (2010)	NA	Physical Activity	Observational—Mixed	Tools and resources created to addressed community-identified issues	Youth aged 8–18 (35)	Federal (IHS^17^)	Yes	Yes
Richards (2012)	Northern Plains	Pregnancy, Preconception Planning	Experimental	Increase in knowledge regarding preconception health, smoking, diabetes, and obesity	Incoming female freshmen (77)	Tribal (Northern Plains Tribal Health Administration Department)	No	Yes
Rink (2012)	Northern Plains	Pregnancy, Prevention	Observational— Qualitative	Increase in knowledge about birth control and the role of mental health on intent to use birth control	Men aged 18–24 (112)	Federal (OPA^19^)	No	Yes
Rushing (2012)	Pacific Coast	STDs/HIV Prevention (Youth)	Observational—Quantitative	Development of technology-based health interventions	Tribal coalition members, youth, topical experts (1271)	Private (NPAIHB^20^)	No	Yes
Smith (2008)	Northern Plains	Cancer, Screening (Cervical)	Observational—Mixed	Increase in knowledge regarding barriers to Pap test screening	Women (101)	Private (ACS^5^)	Yes	Yes
Strickland (2014)	NA	Diabetes, Cancer (Breast)	Curriculum Development	Community research capacity built	Members of 2 tribal communities (NA)	Federal (NINR^21^)	Yes	Yes
Subrahmanian (2011)	Northern Plains	Cancer, Screening (All types)	Curriculum Development	Increase in knowledge regarding cancer etiology and screening	Adults (404)	Federal (NCI^3^)	Yes	No
Teufel-Shone (2006)	Southwest	Wellness (Young Adult)	Observational— Qualitative	Wellness instruments culturally tailored	Adults (48)	Federal (NARCH^22^)	Yes	Yes
Tilburt (2013)	Alaska/Northern Plains	Cancer, Follow-up Care (Prostate)	Observational—Mixed	Relations built to reduce cancer incidence	Males aged 50–80 with a documented PSA test > 4.0 ng/mL (NA)	Federal (CDC^13^)	No	Yes

^1^National Institute on Alcohol Abuse and Alcoholism. NA, not available.

^2^National Institute of Diabetes and Digestive and Kidney Diseases.

^3^National Cancer Institute.

^4^National Institute on Minority Health and Health Disparities.

^5^American Cancer Society.

^6^American Lung Association.

^7^US Department of Health and Human Services.

^8^Health Resources and Services Administration.

^9^National Institutes of Health.

^10^The Robert Wood Johnson Foundation.

^11^National Institute on Drug Abuse.

^12^National Institute for Occupational Safety and Health.

^13^Centers for Disease Control and Prevention.

^14^American Liver Foundation.

^15^National Center for Research Resources.

^16^National Institute of General Medical Sciences.

^17^Indian Health Service.

^18^National Institute of Mental Health.

^19^Office of Population Affairs.

^20^Northwest Portland Area Indian Health Board.

^21^National Institute of Nursing Research.

^22^Native American Research Centers for Health.

### Assessment of community participation

We used the Reliability-Tested Guidelines for Assessing Participatory Research Projects developed by Mercer et al. in 1998 and updated in 2008 (17) to assess community participation. These guidelines, created in response to the growing interest in CBPR and the need to assist funding agencies in assessing the participatory nature of proposals that included a CBPR orientation, are regularly used by funding agencies to assess the degree of participation in studies using a CBPR orientation. They are also used to aid evaluators in assessing the extent to which projects meet participatory research criteria and to assist researchers and community partners in strengthening the participatory nature of their project proposals and funding applications (17). For the purposes of this article, we used the guidelines to assess the documented participation presented in each article and to evaluate how strongly each article adhered to the participatory research criteria presented in the guidelines.

The guidelines outline 4 key domains: 1) Participants and the Nature of Their Involvement; 2) Shaping the Purpose and Scope of the Research; 3) Research Implementation and Context; and 4) Nature of the Research Outcomes (17). Each of the 4 domains includes key measures to assess participation (Table 1). For example, Domain 1 assesses community representation and participation, trust, and management; Domain 2 assesses the development of research questions, application of community knowledge, determinants of community health, and community capacity planning; Domain 3 assesses mutual learning, decision-making, and participation in planning and analysis; and Domain 4 assesses commitment to action, project ownership, and dissemination. In total, the 4 domains assess 25 items that are rated on a 3-point scale with 1 indicating insufficient information to determine whether a guideline has been addressed at all, 2 indicating information is insufficient to determine whether a guideline has been addressed adequately, and 3 indicating that information is sufficient to determine that the guideline has been addressed adequately (17). Therefore, a study scoring the highest for each of the domains would receive a total score of 75.

As tribal communities are unique from many other communities in several ways such as having their own governments, their own unique health systems, and, sometimes, their own IRBs, the language of the guidelines had to be adapted to fit more closely with the sovereignty of tribal nations. For example, several of the items reference intended users with regard to project management or oversight. In tribal communities, projects may be overseen by IHS administrators, tribal IRBs, tribal councils, or tribal research committees. Therefore, item 1e— “Do the researchers and intended users participating in the research process have a formal or informal agreement (verbal or written) regarding management of the project?”—was interpreted to assess whether tribal or IHS IRB agreements, council memoranda of understanding, or some other agreement from tribal leadership was attained.

Two trained research assistants independently reviewed and scored each article. In the first review, each scorer read and highlighted pertinent information about assessing participation. Then, both assistants reviewed each article 4 additional times, scoring them according to 1 of Mercer et al.’s 4 domains. Next, the content from each article was rated by both reviewers for each of the 25 items in Mercer et al.’s guidelines to summarize adherence to the 4 domains of participatory research. Finally, the reviewers compared their scores for each article. In the event of a scoring disagreement, both reviewers re-examined the article together to determine a final score.

## RESULTS

### Study characteristics

A summary for each of the articles reviewed is shown in Table 2. Studies selected for review were published between 1995 and 2016.

#### IHS region

We categorized study locations by using IHS regions, a common way to categorize AI/AN population or community data (18). Five of the studies did not specify a region (11.9%), and only 1 study took place solely in the Alaska Region (2.4%) with an additional study recruiting participants in both the Alaska and the Northern Plains Region (2.4%). Nearly one-third of the studies (28.5%) were conducted solely in the Northern Plains Region. The remaining studies were fairly equally distributed with nearly a fifth of the studies conducted solely in the Southwest Region (19.1%), 4 solely in the Eastern Region (9.5%), and similar numbers in the Pacific Coast Region (11.9%) and the Southern Plains Region (7.1%). An additional 3 studies recruited participants jointly in the Eastern and Southern Plains Regions (7.1%). Finally, a third of the studies took place solely on a reservation (33.3%), a fifth only in rural areas (20.9%), half solely in urban settings (9.5%), and over a fifth in a combination of settings (21.4%). Four studies did not specify a setting (9.5%).

#### Study topic

Although a wide variety of topics were examined among the 42 articles, diabetes, cancer, substance abuse, and tobacco use were most common. Over a quarter of the reviewed studies were reports of cancer studies or interventions (28.5%). An additional fifth (21.4%) of the studies addressed tobacco, alcohol, or drug use; and, finally, 4 articles reported on diabetes research (9.5%).

#### Research design

Study research designs were categorized as curriculum development, experimental, or observational. The vast majority of studies reported an observational design (76.7%; *n* = 33) whereas only 2 studies reported an experimental design (4.7%). The remaining 9 studies (20.9%) involved curriculum development or adaptation.

#### Outcomes

Almost half of the studies (47.6%) reported an increase in knowledge of study participants with an additional fifth (19%) reporting increased community capacity. One-third of the studies (33.3%) reported deliverable outcomes. Examples of deliverable outcomes from the studies included the creation or cultural tailoring of tools or curricula or a positive environmental change. Finally, 5 of the studies (11.9%) reported an increase in screening rates.

#### Research participants

Nearly a quarter of the studies reported on interventions in which youth either were the study population or participated in the research (23.8%), 7 recruited only females (16.7%), and 3 targeted only AI college students (7.1%).

#### Funding source

The majority of studies received only federal funding (e.g., National Institute of Diabetes and Digestive and Kidney Diseases, National Cancer Institute, etc.) for research (61.9%). Over a fifth of the studies received funding solely from private foundations (e.g., American Cancer Society, Susan G. Komen Foundation) or from a combination of federal and private sources (21.4%). The remaining studies either made no mention of funding or received funding either from university grants or tribal or state funding (16.7%).

#### Authorship

Fifteen of the articles (35.7%) identified a tribal partner member as a coauthor. The remaining 27 articles (64.3%) were not coauthored by tribal partners; however, tribal partners were almost always acknowledged.

#### Tribal IRB or equivalent

Nearly three-quarters of the studies (73.8%) reported having approval from a Tribal IRB or IHS IRB, or similar governing body (e.g., tribal council, tribal chief, etc.). The remaining studies either had approval from only a university IRB (19.0%) or made no mention of IRB approval in the article (7.1%).

### Assessment of participatory research

Only 4 of the 42 studies assessed received a perfect score of *3* for each of the 25 questions (9.5%), indicating complete adherence to all guidelines. Likewise, only 2 of the 42 studies scored excessively low (i.e., lower than *2* for each of the 25 questions). The mean score of the studies assessed was 2.7 (SD = 0.32). Over one-third of the articles (35.7%) assessed scored below the mean. Because the scores were not normally distributed, the median was calculated and reported to be 2.8. **Table 3** illustrates the frequencies and percentages for the 25 items that comprise each of the 4 domains for the studies reviewed.

**TABLE 3:** Distribution of study adherence to reliability-tested guidelines for assessing participatory research projects (*n* = 42)

Best practice guideline	Not addressed percentage of total (no.)	Partially addressed percentage of total (no.)	Sufficiently addressed percentage of total (no.)	Adherence score, M (SD)
Domain 1: Participants and the Nature of Their Involvement				
1a: Users described	0 (0)	7.1 (3)	92.9 (39)	2.93 (0.26)
1b: Needs considered	0 (0)	4.8 (2)	95.2 (40)	2.95 (0.22)
1c: Barriers addressed	7.1 (3)	16.7 (7)	76.2 (32)	2.9 (0.60)
1d: Trust built	4.8 (2)	4.8 (3)	90.5 (37)	2.86 (0.47)
1e: Management agreed	4.8 (2)	0 (0)	95.2 (40)	2.90 (0.43)
Domain 2: Shaping the Purpose and Scope of the Research				
2a: Questions developed together	26.2 (11)	19.0(8)	54.8 (23)	2.29 (0.86)
2b: Knowledge applied	2.4 (1)	9.5 (4)	88.1 (37)	2.86 (0.42)
2c: Mutual learning	7.2 (3)	19.0 (8)	73.8 (31)	2.67 (0.61)
2d: Multiple determinants addressed	23.8 (10)	11.9 (5)	64.3 (27)	2.40 (0.86)
2e: Capacity built	4.8 (2)	21.4(9)	73.8 (31)	2.69 (0.56)
Domain 3: Research Implementation and Context				
3a: Knowledge implemented	2.4 (1)	9.5 (4)	88.1 (37)	2.86 (0.42)
3b: Community learning research	14.3 (6)	35.7 (15)	50.0 (21)	2.36 (0.73)
3c: Learning from community	2.4 (1)	9.5 (4)	88.1 (37)	2.86 (0.42)
3d: Decision-making agreement	26.2 (11)	2.4 (1)	71.4 (30)	2.45 (0.89)
3e: Data collection	9.5 (4)	23.8 (10)	66.7 (28)	2.57 (0.67)
3f: Analysis	11.9 (5)	23.8 (10)	64.3 (27)	2.52 (0.71)
3g: Interpreting findings	2.4 (1)	16.7 (7)	80.9 (34)	2.79 (0.47)
Domain 4: Nature of the Research Outcomes				
4a: Commitment to action	2.4 (1)	7.1 (3)	90.5 (38)	2.88 (0.40)
4b: Interpretation agreement	30.2 (13)	0 (0)	69.8 (29)	2.38 (0.94)
4c: Ownership agreement	35.7 (15)	4.8 (2)	59.5 (25)	2.24 (0.96)
4d: Feedback agreement	7.1 (3)	0 (0)	92.9 (39)	2.86 (0.52)
4e: Dissemination agreement	26.2 (11)	0 (0)	73.8 (31)	2.48 (0.89)
4f: Participate in dissemination	20.4 (9)	14.3 (6)	64.3 (27)	2.43 (0.83)
4g: Results applied	0 (0)	7.1 (3)	92.9 (39)	2.93 (0.26)
4h: Sustainability	2.4 (1)	9.5 (4)	88.1 (37)	2.86 (0.42)

#### Domain 1: Participants and the Nature of Their Involvement

The first domain, “Participants and the Nature of Their Involvement,” consists of 5 items that assess community participation, particularly in the formative steps of the research process (e.g., Have community members been adequately described in the article? Have community needs been considered? Have barriers to participation been addressed? etc.). Overall, the studies assessed were more likely to receive perfect scores for each item in Domain 1, compared to the other 3 domains. The majority of all studies fully described the intended users (92.9%), fully considered their needs (95.2%), and had either a formal or informal agreement regarding management of the project (95.2%). Similarly, the majority (88.1%) of the studies assessed provided specific means to building trust among intended users. Addressing barriers was the least addressed item in Domain 1; however, over three-quarters (76.2%) of studies still managed to completely address barriers to participating in research among intended users.

#### Domain 2: Shaping the purpose and scope of the research

The second domain, “Shaping the Purpose and Scope of the Research,” also consists of 5 items. Within this domain were items regarding community involvement in research aims (e.g., Were research questions developed collaboratively and was community knowledge used?) as well as considerations for broader community health impacts (e.g., Has the project provided mutual learning opportunities? Have multiple levels of health determinants been addressed? Has capacity been built to address these broader determinants?). For Domain 2, most of the studies reported applying the unique knowledge of the community (88.1%) with nearly all remaining studies (9.5%) at least partially applying community knowledge to the conceptualization of the research. Almost three-quarters of the studies (73*.8%*) reported making provisions to build the capacity of the intended users with an additional one-fifth (21.4%) reporting moderate plans to build capacity. However, over a quarter (26.1%) of the studies reported that the research question for the study was developed entirely by the researchers with no input from the intended users. Likewise, several of the studies assessed (*n* = 10) considered only 1 layer of the ecological model of health (19) for their studies.

#### Domain 3: Research Implementation and Context

The third domain, “Research Implementation and Context,” consists of 7 items regarding community roles in the execution of research (e.g., Are community knowledge and experience applied in research implementation? Do community members have the opportunity to learn about research methods, data collection, analysis, and interpretation of results? etc.). Nearly a third of the studies (30.9%) received a *3* for all 7 questions in Domain 3, “Research Implementation and Context.” Noticeably, though, some of the studies (26.2%) made no mention of any sort of agreement regarding mutual decision-making with the community members or leadership. These results show that over a quarter of the studies assessed made no mention of how differences of opinion between researchers and community stakeholders would be addressed. Conversely, nearly all (88.1%) studies reported using local knowledge and experience in the implementation of research. Interestingly, most of the studies (88.1%) reported that the academic researchers had substantial opportunity to learn about the perspectives of the community, whereas half (50.0%) of the studies reported that the community had substantial opportunity to learn about research.

#### Domain 4: Nature of the Research Outcomes

The final domain, “Nature of the Research Outcomes,” consists of 8 items regarding research findings (e.g., How will differences in results interpretations be resolved? How will feedback be received? Who owns the data? etc.) and their usage (e.g., Will research findings lead to action?). This domain also aims to assess potential for long-term sustainability that may result from collaboration between the community and resource providers. Sixteen of the studies fully addressed each of the 8 questions asked for Domain 4, “Nature of the Research Outcomes.” Over one-third of the studies (35.7%) made no mention of a formal or informal agreement regarding ownership and sharing of research data. Ownership agreements in AI communities include IRB protocols, memoranda of understanding, or tribal council approval. This question received the lowest mean, 2.3 out of 3.0 for all 25 questions, meaning that ownership was the least addressed item across all 4 domains.

Almost all the studies (90.5%) reported a sufficient commitment to action from both researchers and the community, using the knowledge, skills, or attitudes gained from the research. Even more of the studies (92.9%) demonstrated a high probability of study results being applied in the community. Finally, nearly all the studies (88.1%) outlined plans for sustainability in relation to the purpose of their research.

### Assessment of association between community participation and outcomes

The nature of reported outcomes varied among studies varied. Nearly half of the studies reported an increase in the knowledge of university and community partners about the topic they were studying (44.2%). Another outcome reported by many of the reviewed studies was an increased adherence to one or more of the 9 CBPR principles (9). For example, many articles cited colearning, knowledge transfer, use of community resources, and the establishment of long-term partnerships as primary outcomes. Seven studies reported more concrete outcomes, such as the development of a curriculum or program based on community input; an additional 4 studies either developed or tailored an existing instrument to make the program more culturally appropriate to the community.

Although all articles reported some type of outcome for their study, of the one-third of reviewed studies (26.2%) that reported tangible outcomes (e.g., tools developed or tailored, environments changed, etc.) one-fifth (19.0%) scored higher than the median (2.8) for all articles assessed. Articles that scored higher than the median were more likely to report outcomes that related to the 9 CBPR principles; nearly all outcomes, regardless of score, reported some positive outcome. None of the articles reported a reduction in disease incidence; however, 5 articles reported an increase in disease screening rates.

## Discussion

Even though our sampling strategy included search phrases other than CBPR, all articles assessed mentioned CBPR and its principles. Many of the articles reviewed specifically mentioned both academic and community preference for using a CBPR approach to research. Further, almost all articles presented detailed descriptions of their AI community partners and the specifics of their participation. Domain 1, “Participants and the Nature of Their Involvement,” had the majority (*n* = 42) of studies assessed, scoring top marks for all 5 questions. Very few of the articles reviewed (*n* = 8) reported a failure to address an item for any question. Addressing barriers to participation among under-represented members of the community in the research project was the most common area that studies failed to address sufficiently. The 2 most common reasons why these studies did not sufficiently address barriers was because some studies used a snowball or convenience sample, whereas others had a specific subgroup of the community in mind when conceptualizing the project and, therefore, did not make considerations for other members.

Although all the studies reported outcomes, only 11 were able to report tangible outcomes (e.g., creation or adaptation of a tool, curriculum, or program). For our third aim, we assessed if increased community participation is associated with improved health outcomes within AI communities.

Interestingly, only 2 studies reviewed used an experimental design or conducted a randomized control trial (RCT). RCTs require randomly assigning participants to different study conditions (e.g., intervention groups and control groups). RCTs have been labeled the gold standard of health research (20); however, as the findings of this review indicate, few RCTs use a CBPR methodology. In CBPR research, and particularly within AI communities, the most significant complicating factor in conducting RCTs is the ethical implications of withholding treatment. For example, in a small tribal community with a high prevalence of hypertension, it would be unethical to give only half of the hypertensive community members an antihypertensive drug; and, likely, community leaders would not approve such a design. The 2 studies reviewed that used an experimental design demonstrated that using RCTs in AI communities while using a CBPR methodology is not impossible. Using a CBPR methodology in RCTs, where applicable, is essential for AI populations because it ensures that studies shift from efficacious (i.e., working under highly controlled conditions) to effective (i.e., working in real-world settings) (21).

Mercer et al. (17) recommend that, for evaluating multiple projects, the guidelines should be used both quantitatively and qualitatively. They suggest that quantitative scores be used to identify a general picture of the participatory nature of a project and that the information gleaned should be accompanied by a qualitative write-up that highlights the participatory aspects of the proposal, identified by applying the guidelines. Given that the guidelines are regularly used in this way by funding agencies to assess the degree of participation and by evaluators to assess the extent to which projects meet participatory research criteria, we believe that the guidelines are an appropriate way to assess articles reporting a CBPR study with tribal communities. As the guidelines were not created specifically for tribal communities, however, future research should seek to tailor the language of the guidelines to be more tribal-specific.

Mercer et al.’s guidelines (17) are comparable to Wallerstein and Duran's CBPR Conceptual Model in that both attempt to assess the degree of community participation for projects. As the guidelines are presented in a checklist format, they were selected for use for this systematic review, as they were a simpler way for each reviewer to assess each article independently. However, the use of Wallerstein and Duran's (2010) model may have provided richer data, as their 4 “domains” consisted of interactive toolkits for each item as opposed to closed-ended questions. In addition, the CBPR Conceptual Model (22) included additional items that Mercer et al. did not, such as the percentage of research dollars allotted to community partners and the amount of time spent in the research partnership. As Wallerstein and Duran's (2010) model offers a more in-depth assessment of participation, we agree with Mercer et al. that their guidelines are appropriate for reviewing multiple projects and recommend that the CBPR Conceptual Model (22) be used when reviewers have fewer projects to assess or need a more in-depth analysis.

### Limitations of this assessment

There were challenges in assessing each study for adherence to CBPR principles for several reasons. First, not all the information needed to assess adherence to CBPR principles could be gleaned from the articles as written. In these instances, the study received a score of 1, which was often defined as having insufficient information to assess. For example, a score of 1 could be due to word or page-length limitations of a manuscript, rather than the absence of particular elements in the CBPR process. In addition, publication bias, the nonpublication of studies with negative or null results (23, 24), may have limited the published studies available for our review. In addition, to group studies by IHS region, we excluded non-US articles, thereby excluding First Nations communities as well as Native Hawaiian communities. Finally, because we used a set of guidelines that are not explicit in the definitions of low, medium, or high participation to rank each study, we essentially attempted to quantify qualitative measures. However, Mercer et al. suggested that the best way to use these guidelines when evaluating multiple articles is to use the guidelines both quantitatively and qualitatively. The quantitative ratings can identify a general picture of the participatory nature of a project by identifying how many guidelines overall and within each domain a project scored as most participatory (17). The numbered score should then be interpreted in conjunction with an accompanying qualitative write-up that highlights weaknesses and strengths of specific participatory aspects of the proposal identified by applying the guidelines (17).

### Recommendations for future CBPR research in American Indian Communities

Based on the review of the current literature, there are several key areas that can be improved in future CBPR projects with AI communities. First, half of the articles assessed (50.0%) had research questions that were developed almost entirely by the researchers with little to no input from the AI communities. Seifer argued that, for capacity to be built, community-defined concerns should direct the trajectory of research. She also contended that refining a research question or confirming its validity with community partners adheres to the rigor needed to conduct effective community research (25).

In addition, when publishing future studies, researchers should cite the development of an ownership agreement. Over half (54.8%) of the articles reviewed made no mention of a formal or informal agreement regarding data ownership or data sharing. Formal agreements regarding ownership and data sharing are usually outlined in a research protocol to a tribal IRB or the IRB of a government agency like IHS, whereas other agreements can come from tribal chiefs, tribal councils, or other community leadership.

Reviewing studies involving AI communities using an established tool to assess community participation yielded enlightening results. Using other CBPR assessment tools to understand AI community research could prove informative for future researchers. For example, The CBPR Conceptual Model (1, 22) is a widely used logic model that could easily be applied to the lens of AI research. Further, the use of reliability-tested guidelines to assess the adherence to CBPR principles raised many important questions about the nature of research in AI communities. Although all studies reviewed reported a CBPR methodology, there was a great deal of variance in adherence to all CBPR principles and reported processes among the reviewed studies. Although this is not unique to CBPR with AI communities, improving understanding about differences in and adherence to CBPR principles and how their implementation impacts long-term engagement and CBPR interventions and outcomes among AIs and tribal nations is important for advancing health equity. Lessons learned in these areas are critical for further elucidating and reducing commonly cited barriers to effective CBPR such as mistrust, power inequities, and governance structures (26). Formalization of reporting standards for CBPR methods would be beneficial for evaluator reviewers to glean whether a study used adhered to CBPR principles with AI and other communities.

## Conclusion

Based on our findings, we have determined that although a CBPR orientation yields improved outcomes, we cannot conclude that scoring higher on Mercer's guidelines led to improved health outcomes. It should be noted, however, that nearly three-quarters of the studies reviewed scored higher than the mean of 2.72, meaning that the majority of studies reviewed exhibited a greater commitment to a CBPR research orientation. Researchers should implement and report process evaluation measures to assess, and better evaluate the impact of, adherence to guiding CBPR principles.

## Supplementary Material

nzz008_Supplemental_File

## Notes

This paper summarizes proceedings from the first author's presentation at the First Annual Conference on Native American Nutrition in 2016. These analyses were conducted as part of the first author's doctoral dissertation.

None of the authors report a conflict of interest related to research presented in this article.

Published in a supplement to *Current Developments in Nutrition*. This article appears as part of the supplement “Proceedings of the First and Second Annual Conferences on Native American Nutrition,” sponsored by the Shakopee Mdewakanton Sioux Community's Seeds of Native Health campaign through a gift to the University of Minnesota. The guest editors of the supplement, Treena Delormier and Mindy Kurzer, have no conflicts of interest. The opinions expressed in this publication are those of the authors and are not attributable to the sponsors or the publisher, Editor, or Editorial Board of *Current Developments in Nutrition*.

Online Supporting Material is available from the “Supplementary data” link in the online posting of the article and from the same link in the online table of contents at https://academic.oup.com/cdn/.

Abbreviations used: AI, American Indian; CBPR, community-based participatory research; IHS, Indian Health Service; IRB, Institutional Review Board; RCT, randomized controlled trial.
