# Supplementary material for: Community-Based Participatory Intervention Research with American Indian Communities: What is the State of the Science?
Source: Curr Dev Nutr. 2019 Feb 26;3(Suppl 2):39–52. doi: 10.1093/cdn/nzz008 (PMC6700457; doi:10.1093/cdn/nzz008)
Supplement: nzz008_Supplemental_File [file nzz008_supplemental_file.doc]

Online Supporting Material

Reference list of studies included in final systematic review

1. Arviso V, Welle D, Todacheene G, et al. Tools for Iina (Life): the journey of the Iina curriculum to the glittering world. Am Indian Alsk Native Ment Health Res. 2012;19(1):124-39. doi:10.5820/aian.1901.2012.124
2. Baldwin JA, Rolf JE, Johnson J, Bowers J, Benally C, Trotter RT. Developing culturally sensitive HIV/AIDS and substance abuse prevention curricula for Native American youth. *J Sch Health.* 1996;66(9):322-327.
3. Brown BD, Harris KJ, Harris JL, Parker M, Ricci C, Noonan C. Translating the diabetes prevention program for Northern Plains Indian youth through community-based participatory research methods. *Diabetes Educ.* 2010;36(6):924-935.
4. Christopher S, Gidley AL, Letiecq B, Smith A, McCormick AK. A cervical cancer community-based participatory research project in a Native American community. Health Educ Behav. 2008;35(6):821-34. doi:10.1177/1090198107309457
5. Coe K, Wilson C, Eisenberg M, Attakai A, Lobell M. Creating the environment for a successful community partnership. *Cancer.* 2006;107(8 Suppl):1980-1986.
6. Colclough YY, Brown GM. End-of-life treatment decision making: American Indians' perspective. Am J Hosp Palliat Care. 2014;31(5):503-12. doi:10.1177/1049909113489592, 10.1177/1049909113489592
7. Daley CM, Greiner KA, Nazir N, et al. All Nations Breath of Life: using community-based participatory research to address health disparities in cigarette smoking among American Indians. Ethn Dis. 2010;20(4):334-8. Cited in: Ovid MEDLINE(R) at <http://ovidsp.ovid.com/ovidweb.cgi?T=JS&PAGE=reference&D=med6&NEWS=N&AN=21305818>. Accessed December 17, 2018.
8. Fahrenwald NL, Belitz C, Keckler A. Outcome evaluation of 'sharing the gift of life': an organ and tissue donation educational program for American Indians. Am J Transplant. 2010;10(6):1453-9. doi:10.1111/j.1600-6143.2010.03120.x, 10.1111/j.1600-6143.2010.03120.x
9. Filippi MK, Ndikum-Moffor F, Braiuca SL, et al. Breast cancer screening perceptions among American Indian women under age 40. J Cancer Educ. 2013;28(3):535-40. doi:10.1007/s13187-013-0499-4, 10.1007/s13187-013-0499-4
10. Fleischhacker S, Byrd RR, Ramachandran G, et al. Tools for healthy tribes: improving access to healthy foods in Indian country. *Am J Prev Med.* 2012;43(3 Suppl 2):S123-129.
11. Forcehimes AA, Venner KL, Bogenschutz MP, et al. American Indian methamphetamine and other drug use in the Southwestern United States. Cultur Divers Ethni Minor Psychol. 2011;17(4):366-76. doi:10.1037/a0025431, 10.1037/a0025431
12. Gilder DA, Luna JA, Roberts J, et al. Usefulness of a survey on underage drinking in a rural American Indian community health clinic. Am Indian Alsk Native Ment Health Res. 2013;20(2):1-26. doi:10.5820/aian.2002.2013.1
13. Goins RT, Garroutte EM, Fox SL, Dee Geiger S, Manson SM. Theory and practice in participatory research: lessons from the Native Elder Care Study. Gerontologist. 2011;51(3):285-94. doi:10.1093/geront/gnq130, 10.1093/geront/gnq130
14. Guadagnolo BA, Petereit DG, Helbig P, et al. Involving American Indians and medically underserved rural populations in cancer clinical trials. *Clin Trials.* 2009;6(6):610-617.
15. Haozous EA, Eschiti V, Lauderdale J, Hill C, Amos C. Use of the talking circle for Comanche women's breast health education. J Transcult Nurs. 2010;21(4):377-85. doi:10.1177/1043659609360847, 10.1177/1043659609360847
16. Hartmann WE, Gone JP. Incorporating traditional healing into an urban American Indian health organization: a case study of community member perspectives. J Couns Psychol. 2012;59(4):542-54. doi:10.1037/a0029067, 10.1037/a0029067
17. Helitzer D, Willging C, Hathorn G, Benally J. Building community capacity for agricultural injury prevention in a Navajo community. *Journal of agricultural safety and health.* 2009;15(1):19-35.
18. Horn K, McCracken L, Dino G, Brayboy M. Applying community-based participatory research principles to the development of a smoking-cessation program for American Indian teens: "telling our story". *Health Educ Behav.* 2008;35(1):44-69.
19. Jernigan VB, Salvatore AL, Styne DM, Winkleby M. Addressing food insecurity in a Native American reservation using community-based participatory research. Health Educ Res. 2012;27(4):645-55. doi:10.1093/her/cyr089, 10.1093/her/cyr089
20. Katz JR, Martinez T, Paul R. Community-based participatory research and American Indian/Alaska Native nurse practitioners: a partnership to promote adolescent health. J Am Acad Nurse Pract. 2011;23(6):298-304. doi:10.1111/j.1745-7599.2011.00613.x, 10.1111/j.1745-7599.2011.00613.x
21. Lopez ED, Sharma DK, Mekiana D, Ctibor A. Forging a new legacy of trust in research with Alaska Native college students using CBPR. Int J Circumpolar Health. 2012;71:18475. doi:10.3402/ijch.v71i0.18475, 10.3402/ijch.v71i0.18475
22. Lowe J, Liang H, Riggs C, Henson J, Elder T. Community partnership to affect substance abuse among Native American adolescents. Am J Drug Alcohol Abuse. 2012;38(5):450-5. doi:10.3109/00952990.2012.694534, 10.3109/00952990.2012.694534
23. Markus SF. Photovoice for healthy relationships: community-based participatory HIV prevention in a rural American Indian community. Am Indian Alsk Native Ment Health Res. 2012;19(1):102-23. doi:10.5820/aian.1901.2012.102
24. Matloub J, Creswell PD, Strickland R, et al. Lessons learned from a community-based participatory research project to improve American Indian cancer surveillance. Prog. community health partnersh.. 2009;3(1):47-52. doi:10.1353/cpr.0.0058, 10.1353/cpr.0.0058
25. Mendenhall TJ, Berge JM, Harper P, et al. The Family Education Diabetes Series (FEDS): community-based participatory research with a midwestern American Indian community. Nurs Inq. 2010;17(4):359-372.
26. Montgomery M, Manuelito B, Nass C, Chock T, Buchwald D. The Native Comic Book Project: native youth making comics and healthy decisions. J Cancer Educ. 2012;27(1 Suppl):S41-6. doi:10.1007/s13187-012-0311-x, 10.1007/s13187-012-0311-x
27. Mullany B, Barlow A, Goklish N, et al. Toward understanding suicide among youths: results from the White Mountain Apache tribally mandated suicide surveillance system, 2001-2006. Am J Public Health. 2009;99(10):1840-8. doi:10.2105/AJPH.2008.154880, 10.2105/AJPH.2008.154880
28. Mullany B, Barlow A, Neault N, et al. The Family Spirit trial for American Indian teen mothers and their children: CBPR rationale, design, methods and baseline characteristics. *Prevention science: the official journal of the Society for Prevention Research.* 2012;13(5):504-518.
29. Ndikum-Moffor FM, Braiuca S, Daley CM, Gajewski BJ, Engelman KK. Assessment of mammography experiences and satisfaction among American Indian/Alaska Native women. Womens Health Issues. 2013;23(6):e395-402. doi:10.1016/j.whi.2013.08.003, 10.1016/j.whi.2013.08.003
30. Noe TD, Manson SM, Croy C, McGough H, Henderson JA, Buchwald DS. The influence of community-based participatory research principles on the likelihood of participation in health research in American Indian communities. *Ethn Dis.* 2007;17(1 Suppl 1):S6-14.
31. Novins DK, Boyd ML, Brotherton DT, et al. Walking on: celebrating the journeys of Native American adolescents with substance use problems on the winding road to healing. J Psychoactive Drugs. 2012;44(2):153-9. doi:10.1080/02791072.2012.684628
32. Nsiah-Kumi PA, Lasley S, Whiting M, et al. Diabetes, pre-diabetes and insulin resistance screening in Native American children and youth. *Int J Obes (Lond).* 2013;37(4):540-545.
33. Palacios JF, Strickland CJ, Chesla CA, Kennedy HP, Portillo CJ. Weaving dreamcatchers: mothering among American Indian women who were teen mothers. *J Adv Nurs.* 2014;70(1):153-163.
34. Perry C, Hoffman B. Assessing tribal youth physical activity and programming using a community-based participatory research approach. Public Health Nurs. 2010;27(2):104-14. doi:10.1111/j.1525-1446.2010.00833.x, 10.1111/j.1525-1446.2010.00833.x
35. Richards J, Mousseau A. Community-based participatory research to improve preconception health among Northern Plains American Indian adolescent women. Am Indian Alsk Native Ment Health Res. 2012;19(1):154-85. doi:10.5820/aian.1901.2012.158
36. Rink E, FourStar K, Medicine Elk J, Dick R, Jewett L, Gesink D. Pregnancy prevention among American Indian men ages 18 to 24: the role of mental health and intention to use birth control. Am Indian Alsk Native Ment Health Res. 2012;19(1):57-75. doi:10.5820/aian.1901.2012.57
37. Rushing CS, Stephens D. Tribal recommendations for designing culturally appropriate technology-based sexual health interventions targeting Native youth in the Pacific Northwest. Am Indian Alsk Native Ment Health Res. 2012;19(1):76-101. doi:10.5820/aian.1901.2012.76
38. Smith AJ, Christopher S, LaFromboise VR, Letiecq BL, McCormick AK. Apsaalooke women's experiences with Pap test screening. *Cancer Control.* 2008;15(2):166-173.
39. Strickland CJ, Hillaire E. Conducting a Feasibility Study in Women's Health Screening Among Women in a Pacific Northwest American Indian Tribe. *J Transcult Nurs.* 2016;27(1):42-48.
40. Subrahmanian K, Petereit DG, Kanekar S, et al. Community-based participatory development, implementation, and evaluation of a cancer screening educational intervention among American Indians in the Northern Plains. J Cancer Educ. 2011;26(3):530-9. doi:10.1007/s13187-011-0211-5, 10.1007/s13187-011-0211-5
41. Teufel-Shone NI, Siyuja T, Watahomigie HJ, Irwin S. Community-based participatory research: conducting a formative assessment of factors that influence youth wellness in the Hualapai community. *Am J Public Health.* 2006;96(9):1623-1628.
42. Tilburt JC, James KM, Koller K, et al. Assessing follow-up care after prostate-specific antigen elevation in American Indian / Alaska Native Men: a partnership approach. Prog. community health partnersh.. 2013;7(2):153-61. doi:10.1353/cpr.2013.0019, 10.1353/cpr.2013.0019
